# Editorial Notes, News and Miscellany

**Published:** 1913-02

**Authors:** 


### Editorial Notes, News and Miscellany.

The Review of Dr. Auld's remarkable book, published under "Book Notices," will be found of unusual interest to physicians. It is new and, I may say, startling. Read it.

---

Death of Dr. Stuart.—Dr. J. R. Stuart of Houston, president of the Houston Infirmary, one of the most prominent physicians of that city, was instantly killed in an automobile accident January 12.

---

A Lunacy Commission.—A bill is before the Texas Legislature to appoint three physicians in each county as a commission to pass upon all cases of suspected or accused lunacy. This would take them out of the court where they are dealt with as criminals. The "Red Back" has "harped" on this for twenty years. It has come at last.

---

TERMS THAT MAKE ME TIRED.—"The busy practitioner," "A complication of diseases," "His lungs were involved," "Heart failure," "Nourishment," "I put her on" so and so. "Iodide of potash."

---

"THE BEST WHAT AM"—the "RED BACK." So says Dr. C. E Hall, Lindale, in the language of William Green Hill, familiarly known as Billy. The occasion for the remark was a fiver which sets his sub up to 1917.

---

SEMICENTENNIAL of Farbwerke-Hoechst Company, New York, successors to Victor Koeche Company. This well-known firm, from a small beginning, has grown, until, at the time of reaching their half century mark in January, they now employ 12,000 people. Our friend, Dr. H. S. Baketel, formerly with the Denver Chemical Company, is their advertising manager, and, of course, the firm is represented in our advertising pages.

---

LIMERICKS (the "RED BACK'S" own)—

Fastidious was Dr. Carlisle; he dressed in the nobbiest stysle; if a lady he'd meet, in crossing the street, he'd dextrously doff his new tisle.

There was an old doctor named Talliafero (Tolliver); he hailed from up about Bolliafero; he drove an odd pair—a horse and a mare; and his "cub" was a young fellow named Olliafero. (Next!)

---

A GALLANT young captain was called up by his colonel to explain assaulting the sentry on his return to the barracks after a dinner on the previous night. The captain had forgotten the incident entirely. The sentry declared that the captain was drunk. The captain's Irish soldier servant, however, emphatically protested that his master was sober. "How is it that you are so sure that he was sober?" asked the colonel; "did he speak to you?" "He did, sorr." "What did he say?" "He tould me to be sure and call him early in the morning, sorr." "That seems all right," said the colonel; "and did—ah—did the captain say why he wished to be called early?" "He did, sorr; he said he was going to be queen of the May, sorr."—(Swiped.)

DON'T—Plato wisely said: To abstain at the right moment is as productive of success as to perform. Man profits by experience —or should do so—and it is almost unpardonable to make the same mistake twice.

The Fellows Company, of New York, manufacturers of the world-famous Fellows Compound Syrup of Hypophosphites, has collected, from many sources, mistakes made by physicians who have recorded them, and in a neat pamphlet, a copy of which can be had free on request, has presented a catalog of "Dont's" in every branch of practice, medical and surgical. They serve as beacon lights to warn against hidden rocks—mistakes that have been made by others. These warnings will surely be of use to the physician in his daily practice. Ask for it, mentioning the "RED BACK."

---

DR. FRIEDMAN'S "SURE CURE" FOR CONSUMPTION.—The United States Department of State asked Consul General Thackara at Berlin to give the department all the information he could obtain on the subject of this alleged discovery. When the consul's report reached Washington, Senator Gore had a resolution passed calling for it to be submitted to the Senate. Here is Dr. Friedman's letter to Consul Thackara. It doesn't amount to a row of pins, and leaves us as wise as before:

"My remedy for the time being has not yet been given out to any one. For the present patients will be treated only under my personal direction in my institute for tuberculosis and scrofulosis at 49 Lutzowstrasse, Berlin. I am unable to say just how soon my remedy will be available in America.

"My institute is not a hospital, but room and board may be had elsewhere in Berlin at usual prices by those who come for treatment.

"It is impossible to give an estimate of length of time necessary for treatment, without examination. Where cases are not so far advanced, treatment usually covers a period of several weeks."

The reluctance to permit other physicians to try the alleged "cure" looks suspicious and much like quackery. Guthrie gave chloroform to the profession, Koler gave away cocaine, and Long, ether, none thought of making money out of their great discoveries.

---

HOLOTHEOL PRODUCTS BID FOR FAVOR UNDER DR. C. EVERETT FIELD—Scientific or business achievement frequently pay but small attention to personality, because, unfortunately, there is but small sentiment in either. At times, however, the condition is changed and friendship holds its count. Dr. C. Everett Field, for many years head of the advertising department of Kress & Owen Company and newly elected as president of the Holotheol Chemical Company of New York, is one of the few who has championed the cause of sentiment in business, and as a result there are a host of friends in both medical and drug circles who are wishing him every success in his new department of effort. Many years' experience in private practice and laboratory work, coupled with his knowledge of the drug business particularly fit him in directing the course of pharmaceutical specialties compounded for physicians' use. Holotheol products are all allied to antiseptics; their formula originating with Dr. Field and every phase of compounding being under his personal supervision, will at all times assure definite and dependable therapy.

Besides being connected with many educational and civic bodies, Dr. Field is a member of the American Medical Association, New York State Medical Society, Queens and Nassau County Medical Societies, National Geographical Society, Advertisers' League, etc. He is president and founder of the People's Forum and organizer of Temple Forum and many societies of the "uplift" kind among young men.

